# Immunodiagnosis — the promise of personalized immunotherapy

**DOI:** 10.3389/fimmu.2023.1216901

**Published:** 2023-07-13

**Authors:** Renjie Wang, Kairong Xiong, Zhimin Wang, Di Wu, Bai Hu, Jinghan Ruan, Chaoyang Sun, Ding Ma, Li Li, Shujie Liao

**Affiliations:** ^1^ Department of Obstetrics and Gynecology, Cancer Biology Research Center, Tongji Hospital, Tongji Medical College, Huazhong University of Science and Technology, Wuhan, China; ^2^ Division of Endocrinology and Metabolic Diseases, The First Affiliated Hospital of Zhengzhou University, Zhengzhou, China

**Keywords:** immunodiagnosis, cancer, immunotherapy, precision medicine, personalized therapy

## Abstract

Immunotherapy showed remarkable efficacy in several cancer types. However, the majority of patients do not benefit from immunotherapy. Evaluating tumor heterogeneity and immune status before treatment is key to identifying patients that are more likely to respond to immunotherapy. Demographic characteristics (such as sex, age, and race), immune status, and specific biomarkers all contribute to response to immunotherapy. A comprehensive immunodiagnostic model integrating all these three dimensions by artificial intelligence would provide valuable information for predicting treatment response. Here, we coined the term “immunodiagnosis” to describe the blueprint of the immunodiagnostic model. We illustrated the features that should be included in immunodiagnostic model and the strategy of constructing the immunodiagnostic model. Lastly, we discussed the incorporation of this immunodiagnosis model in clinical practice in hopes of improving the prognosis of tumor immunotherapy.

## 1 Introduction

The immune system is an interacting network of immune cells, the molecules they produce, and the lymphoid organs that organize these components (1). Proper immune system function is essential for health, and insufficient immune system activity can lead to different types of diseases included tumor.

​In recent years, immunotherapy has yielded new wave in treating tumors with brand-new methods such as immune checkpoint inhibitors (ICIs), adoptive cell therapy (ACT), and therapeutic vaccines. Some patients with tumor types that were previously considered refractory (2) or advanced/metastatic tumors (3) were controlled after receiving ICI treatment. However, most patients do not benefit from immunotherapy (4). In addition, immunotherapy empower immunity against cancer and may lead to immune-related adverse effects (irAEs) such as colitis, dermatitis, pneumonia, and thyroiditis (5). The efficacy and toxicity of immunotherapy remains poorly predictable for given patients till now.

Why do patients with the same disease get dramatically different outcomes when given the same immunotherapy? And how is it possible to tell if a patient might benefit from immunotherapy? Since immunotherapy acts on a strongly heterogeneous immune system of the patient, immune status may be a critical bridge connecting the patient’s characteristics to the outcome of immunotherapy. Therefore, it is reasonable to diagnose the immune state of tumor patients before taking immunotherapy - we pioneering name it as immunodiagnosis (ID). We define immunodiagnosis (ID) as systematically, comprehensively, and dynamically evaluating the status of an individual’s immune system, to reflect at different disease stages the systemic and local immune status. ID could help clinicians judge the disease phenotypes, evaluate disease activity, and predict the possible progress of disease and then develop a personal treatment plan, rather than directly giving “one-size-fits-all” immunotherapy to patients with very different immune status. With ID, clinicians can qualitatively or quantitatively predict possible immune responses of the local and peripheral immune systems to endogenous and exogenous stimuli, thereby guiding medical decisions. The ID idea has found its way into clinical practice. For example, the FDA has approved the expression of PD-L1 as a biomarker to predict how patients with tumors will respond to ICIs. However, currently used models consist of only a single target or a very small number of targets from a single test sample, which does not fully reflect the complexity of the interaction between the immune system and the host in real-world situations and is therefore less efficient to detect.

Based on the existing research, how should the ID model be constructed? An adult has relatively stable baseline levels of immunity (6), and the composition and function of the immune system are heterogeneous among people of different ages (7), sexes (8), or races etc. As an important guardian of human health, the immune system is continuously stimulated by endogenous and exogenous factors, which can cause fluctuations in the immune status, reflected in the number and composition of immune cell groups, response to stimuli, and cytokine levels. At the same time, the fluctuations of the immune system, stimulated by a diverse array of physiological and pathological processes, should not be neglected. To be more specific, when it comes to certain diseases and treatments, there are certain biomarkers that reflect the relevant immune status. Based on the elaboration of the prognostic factors of immunotherapy in previous literature, we believe that ID models should contain multi-dimensional indicators, including patient demographic characteristics which could basically stratify patients into relatively stable groups, health status which could cause fluctuation of the immune system, and some specific biomarkers that are directly related to the mechanism of disease progression or immunotherapy.

It is difficult for human experts to identify hidden associations from such complex and large datasets. Fortunately, artificial intelligence (AI) has the ability to find unstructured features in such datasets that are large (containing a large number of samples) and complex (each sample has many features). In recent years, AI, especially machine learning and deep learning, has been widely applied in disease clinical research, leading to remarkable predictive performance. Studies have reported that traditional analysis methods, such as statistical analysis and multivariate analysis, are less accurate compared to AI, especially when AI is combined with bioinformatics tools to significantly enhance the accuracy of disease diagnosis and prognosis assessment (9, 10).

**Figure 1:**
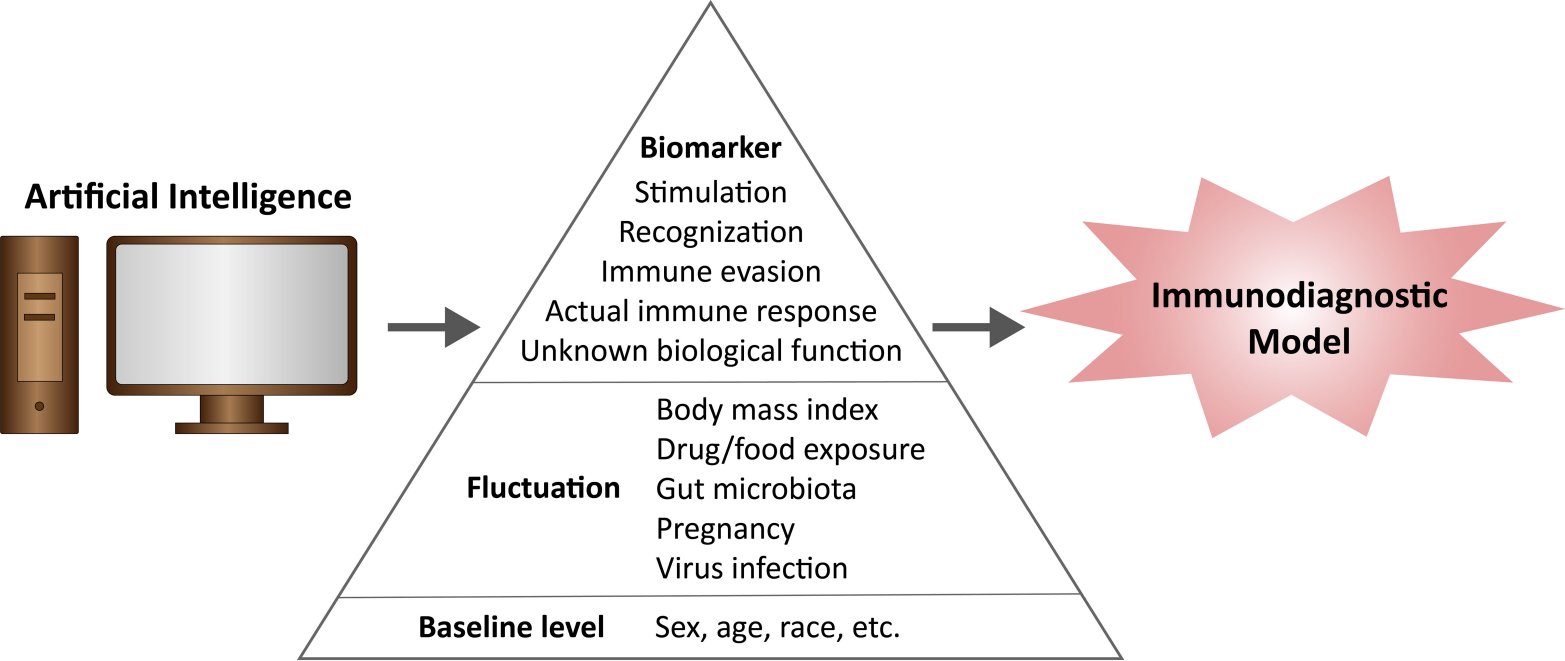
The composition of Immunodiagnositic model.

In this paper, we present for the first time the important concept of ID, provide a preliminary blueprint for ID systems, analyze what features should be included in ID models, and discuss how to construct ID systems based on existing research (as shown in **Figure 1**). Furthermore, we look forward to the application of AI in the construction of ID systems, which may shed light on the realization of tumor-precision immunotherapy.

## 2 Baseline of ID: population stratification of immune status

The immune status can maintain a relatively stable state for several years for an individual (6). However, some intrinsic demographic characteristics are associated with immune status. It is necessary to initially stratify the entire population based on these characteristics and establish a baseline for the ID of different subgroups.

### 2.1 Sex

The prevalence and types of immune disorders vary significantly between males and females. In general, women tend to have a stronger immune response to external and internal stimuli, and are more susceptible to generating antibodies and suffering adverse side effects (11). Cancer rates in 2018 were approximately 1.15 times higher in men compared to women, and deaths from cancer were higher in men than in women (12).

As a potential prerequisite for ID, the immune status of males and females showed significant differences. The researchers analyzed the global immune cell composition of 49 men and 52 women and found that women had higher naive CD4+T cells, while men had higher activated CD8+T cells (13). Single-cell transcriptome analysis of immune cells from peripheral blood revealed a higher proportion of NK cells in men than in women, while GO analysis revealed higher levels of T and B cell activation signaling in women than in men (14). Therefore, the cut-off for these immunological features in ID should be sex-specific.

While sexual disparities in immune status are widely recognized, little is known about how sex affects the efficacy and toxicity of immunotherapies. A meta-analysis examining 7133 studies found that only 20 randomized controlled trials of ICIs reported an OS relationship with sex (15). Among 11351 pan-cancer patients (with melanoma and non-small cell lung cancer (NSCLC) being the most common types), the hazard ratio (HR) of OS in ICIs groups versus control groups was 0.72 in males and 0.86 in females, revealing a better efficacy of ICIs in males compared to females. Nonetheless, there is potential gender discrimination when patients are enrolled, as the number of female patients is less than half of the male patients, thereby skewing the overall data pool. Consequently, the representation and reliability of female data may be inadequate. The sex disparity in the efficacy of immunotherapy varies with disease type. In NSCLC, anti-PD-1/PD-L1 is more effective in women, whereas in colorectal cancer, it is more effective in men (16).

Sex has also been linked to adverse events (AEs) following immunotherapy. Consistent with higher rates of autoimmune disease, women treated with ICIs had more severe AEs, with a 49 percent higher risk than men (17). As a result, women are more likely to discontinue treatment, resulting in a poorer prognosis. Together, these pieces of evidence support the need for a sex-related ID.

### 2.2 Age

Aging is associated with several immune pathologies. The incidence of cancer increases with age as the genetic mutation risk accumulates (18). As age increases, the ability of B cells to produce specific antibodies decreases, but their ability to produce autoantibodies increases (19). Immunosenescence, defined as the function of immune system decreases and the composition of immune system remodeling with age (20), includes increased immune memory cells, decreased bone marrow, decreased antigenic diversity of immune cells, decreased co-stimulatory molecules on T cells, and changes of several inflammatory mediators (IL-1a, IL-8, CRP, etc.) (21). However, it is still unclear how immunosenescence affects the efficacy and safety of immunotherapy.

For ICIs, as demonstrated by studies in glioblastoma (22), NSCLC (23), and hepatocellular carcinoma (24, 25), older patients aged 65 to 75 do not respond worse to ICI treatment than younger patients. In other cases, old age has been shown to be a predictor of better efficacy in immunotherapy. In NSCLC, a benefit has been reported in patients over the age of 70 or 75 (26). In metastatic melanoma, a cohort analysis of 538 metastatic melanoma patients found that anti-PD-1 antibodies were more effective in patients over the age of 60 (27). Additionally, researchers have tested this correlation in preclinical models. With transplanted genetically identical tumors, aged mice (52 weeks) performed stronger response to anti-PD-1 than young mice (8 weeks). This phenomenon may be related to the higher Tregs in young mice (27). Fortunately, the toxicity of immunotherapy has not increased in older patients, as supported by anti-PD-1/PD-L1 and anti-CTLA-4 studies (26, 28). For CAR-T, the advantage of immunotherapy in older patients is supported by a clinical trial that in large cell lymphomas patients, the response rates of elders and youngsters are comparable, but the elders have higher rate of complete responses (62% versus 46%) (29).

However, when the age cutoff is reached at 75 years, several studies have reported a trend towards ICI resistance in patients older than 75 years (30). Nonetheless, the age disparity depends on the status of the individual with the disease or on the different types of disease. A retrospectively study collected data from 254 patients with metastatic melanoma, and divided patients into 4 groups by age (≤50, 50-64, 65-74, ≥75 years), revealing no significant difference in median overall survival (mOS), progression-free survival (PFS) and immune-mediated toxicities among these groups (28). Older patients tend to be excluded from the cohort due to higher levels of underlying disease and complications, so data on immunotherapy in older patients is relatively limited.

### 2.3 Race and ethnicity

Racial ethnical disparities exist in the incidence, mortality, and access to immunotherapy of tumor (31, 32). Furthermore, studies have revealed variations in the normal range of a subset of lymphocytes in people of different races or ethnicities. Indians have higher levels of CD3+ T cells, CD4+ T helper cells, and CD19+ B cells than Chinese or Malays (33). Caucasian Americans have higher levels of γδT cells than African Americans (34). TH17/TH22 is upregulated in Asian patients, while TH17/TH1 is absent in African American patients (35). In addition, signaling activation of immune cells varies across ethnic groups. Single-cell network profiling analysis of a broad panel of immune signaling pathways in peripheral blood mononuclear cell (PBMC) subsets from 60 healthy donors, and found that African Americans had lower B cell anti-IgD-induced pathway activity, including PI3K, MAPK and NF-kB pathway, compared to European Americans (36). These evidences suggest that there are racial differences in immune status and support race as a baseline stratification factor for ID.

There are substantial evidences that race or ethnicity is associated with the outcome of traditional treatments such as chemotherapy for cancers (37), but few studies focused on immunotherapy. It appears that race or ethnicity may be a predictor of efficacy and/or toxicity of immunotherapy, but depends on the treatment strategy.

For ICIs, an observational study enrolled 1,135 patients with unresectable or advanced melanoma treated with anti-PD-1 drugs from 5 institutions in USA, Australia and China, and revealed that white patients have higher overall response rates (ORR) and longer PFS than East Asian, Hispanic, and African (38). As for irAEs, white patients tended to present gastrointestinal irAEs, while other patients had higher rates of endocrine and liver irAEs. Another retrospective cohort of 249 patients with advanced NSCLC treated with anti-PD-1/PD-L1 found that African-American patients had longer treatment discontinuities and longer OS than white patients. The disease control rate was also higher (59.6% versus 56.5%) in African Americans than in white patients (39).

For CAR-T, a retrospective analysis of five Phase I clinical trials involving a total of 139 patients with hematologic malignancies treated with CD19 CAR-T cells found that Hispanic patients were more likely to have severe cytokine release syndrome (40).

For therapeutic vaccines, sipuleucel-T is an autologous cellular vaccine developed for the treatment of asymptomatic/minimally symptomatic metastatic castration-resistant prostate cancer. An observative study involving 1902 patients with prostate cancer treated with sipuleucel-T revealed that the HR of OS between African American and Caucasian is 0.81 (95% CI: 0.68-0.97). African Americans’ superior response to immunotherapy may stem from their higher neoepitopes, which can be recognized by the immune system (41).

Still, the disparity appears to vary by disease type and treatment strategy. A study of patients with triple-negative breast cancer treated with anti-PD-L1 combined with neoadjuvant chemotherapy and reported a trend of lower pathologic complete response (43% versus 48%) and lower three-year event-free survival (71.4% versus 78.3%) in African American patients compared with others, although with no statistical significance (42). Together, these pieces of evidence point to the justification of using race and ethnicity as stratification factors in ID. However, the types of diseases and treatment strategies covered by existing studies are insufficient, and most studies only present clinical information without matching serological information to assess immune status.

## 3 Fluctuations: health states regulate ID

Variant physiological or pathological status can also cause fluctuations in immune status based on baseline immunity levels after stratification of patients by their intrinsic demographic characteristics. We collated the characteristics of immune status and corresponding immunotherapy outcomes in several typical physiological and pathological status.

### 3.1 Body mass index

Obesity (BMI≥30kg/m2 according to WHO standard definition) can promote inflammation and affect the distribution and abundance of immune cells, and has been validated to relate with the process of malignancy (43). Recently, obesity has been shown to be associated with response to immunotherapy. A meta-analysis of 13 eligible studies involving 5,279 patients with pan-cancer treated with ICIs revealed that high BMI was associated with improved PFS and OS (44), and this finding was also validated in a multi-center clinical trial of patients with NSCLC treated with ICIs (45). In contrast, a study involving 181 patients with advanced NSCLC treated with second-line ICI after first-line chemotherapy had failed found that lower BMI was associated with longer PFS and OS (46). Some studies have found that obesity enhances immunotherapy outcomes only in a subgroup of patients. A randomized controlled trial included 207 melanoma patients treated with anti-CTLA-4 plus chemotherapy, as well as one retrospective cohort with 331 melanoma patients treated with anti-PD-1/PD-L1 monoantibodies, also corroborated the positive correlation between obesity and prolonged PFS and OS, and the association was mainly seen in male patients, while no significant difference was observed in female patients (47). Whether the association varies by sex needs further study. Moreover, treatment settings may affect the benefits of obesity. A multicenter study of NSCLC also found obesity to benefit the efficacy of anti-PD-1/PD-L1 antibodies, but only with the setting of ICI as second- or later-line therapy, with no such difference in the cohort with high PD-L1 expression (≥50%) and treated with ICIs as first-line therapy (48). However, a multi-center trial found the high PD-L1 expression subgroup represent the strongest association between BMI and PFS and OS when received ICI as the first-/second-/later-line therapy (45).

Low BMI may indicate cachexia, defined as a body weight loss >5% over the past 6 months or >2% in patients with a BMI< 20 kg/m2, which was common in advanced cancer (49). Not surprisingly, low BMI was associated with poorer clinical outcomes in several studies involving pan-cancer patients treated with ICIs (50). Consistently, cachexia was also associated with worse outcomes (51).

The correlation between the incidence of treatment-related adverse events and BMI is under debate. A meta-analysis of 20 studies designed to reveal associations between irAEs and BMI in pan-cancer patients treated with immunotherapy found a positive association between BMI and higher risk of irAEs (52), and another multicenter retrospective observational study involving 1,070 patients reported the same propensity (53). Nonetheless, a meta-analysis suggested that there was no significant difference in the incidence of all grades of IAEs among obese, overweight, and normal patients (44), and a clinical trial involving 2,110 patients with advanced NCSLC also supported this view (45). For CAR-T therapy, a study included 64 patients receiving CD-19 CAR-T for relapsed/refractory B cell malignancies and found that patients with ≥2 and earlier stage of cytokine release syndrome possessed a significantly higher BMI (54).

The mechanism explaining the predictive effect of BMI remains unclear, as most studies do not distinguish between the amount of skeletal muscle and the amount of adipose tissue, which have completely different biological functions. A more careful investigation is required. One study involved 74 pre-treated NSCLC patients treated with anti-PD-1 therapy and used CT to assess skeletal muscle, visceral adipose, and subcutaneous adipose (55). They found that neither the visceral-to-subcutaneous ratio of adipose nor the visceral fat area was associated with the efficacy of ICI therapy, suggesting that adipose tissue may not influence clinical outcomes. However, they reported that lower intramuscular adipose tissue content was a prognostic factor of longer OS, but was not significantly associated with PFS. Another retrospective study found a correlation between lower muscle mass and worse OS in NSCLC patients treated with ICIs in combination with chemotherapies (56). The predicted values for the mass and adipose content of skeletal muscles need to be further verified.

### 3.2 Exposure to drugs and food

Certain drugs have been observed to be associated with immunotherapy outcomes. A classic example is acetaminophen (APAP), which is widely used to manage mild-to-moderate pain caused by advanced tumors, is suggested to have negative immunomodulatory effects. For ICI therapy, a clinical study involving three separate cohorts found that APAP exposure was significantly associated with worse ORR, OS, and PFS in patients treated with ICIs for advanced renal cell carcinoma (57). The underlying mechanism may be that APAP induces Tregs amplification and penetration into the TME and upregulates the expression of the immunosuppressive molecule IL-10, thereby mediating immunosuppressive effects and reducing the efficacy of immunotherapy (57).

Antibiotics have also been reported in relation to immunotherapy. A meta-analysis included 5,560 NSCLC patients treated with ICIs from 23 studies, revealing that the exposure to antibiotics around ICIs initiation (-60 days, +60days) could dramatically decrease the PFS and OS (58). The analysis demonstrated that the median OS decreased by 6.7 months in the patients exposed to antibiotics. However, strong heterogeneity in treatment-line settings and patient clinical data across studies resulted in weak reliability of the analysis. The mechanisms explaining the effects of antibiotics on the efficacy of immunotherapy remain unclear. These drugs should be used with caution in patients receiving immunotherapy. Whether this principle applies to the onset or entire duration of immunotherapy and to all immunotherapy regimens such as therapeutic vaccines and CAR-T requires further study.

Probiotics are a large category of healthcare products and have emerged as a beneficial complement during immunotherapy. A trial surveyed the dietary habits and probiotics intake of 158 patients with late-stage melanoma, 49 of 158 patients reported probiotic usage in 1 month before the initiating of ICI therapy. The study observed a correlation between probiotics and a significantly reduced frequency of tumor-infiltrating IFN-γ positive CD8+ T cells, as well as fewer tumor-infiltrating TH1 cells though not reach significance, revealing a suppression of anti-tumor immunity caused by probiotics (59). In contrast, other previous studies have found that probiotics may benefit the efficacy of immunotherapy in mouse models and clinical patients. Yusuke Tomita and colleagues retrospectively surveyed 118 NSCLC patients treated with ICIs in Japan and found that probiotic Clostridium difficile therapy was associated with prolonged PFS and OS, even in patients with antibiotic exposure (60). To identify whether diet change in the onset of ICI can safely and effectively improve the clinical outcomes, Christine N Spencer and colleagues are performing a phase II trial (NCT04645680).

### 3.3 Gut microbiota

The gut microbiome can have a systemic effect on the immune system. It has been reviewed that gut microbiome plays an important role in cancer development, anti-tumor immunity, and response to therapy (61). More recently, the gut microbiome has emerged as a predictor of response to immunotherapy. For ICI therapy, studies have shown that specific bacteria can stimulate the immune system and have been demonstrated to augment the efficacy of ICI therapy in mouse models (62, 63). Specifically, Bifidobacterium may improve the activation of DCs and tumor-specific CD8+T cell responses (63), while B fragilis may increase the activation of TH1 cells (62). For adoptive T cell therapy, higher abundance of the Bacteroidales S24-7 family is correlated with higher IL-12 and more CD8α+ DCs in the peripheral blood of mouse model, suggesting that this species could improve anti-tumor immunity (64).

Clinical outcomes also vary depending on the gut microbiome composition. A prospective study enrolled 70 Japanese patients with advanced NSCLC and treated them with anti-PD-1/PD-L1 monoclonal antibodies and performed 16S rRNA sequencing of stool samples. Lower alpha-diversity of gut microbes at baseline was associated with worse OS. Besides, Ruminococcaceae UCG13 and Agathobacter were enriched in patients with reassuring ORR and PFS (65). In contrast, in another clinical trial involved 438 melanoma patients, the alpha and beta diversity of the gut microbiota have no significant differences between ICI responders and nonresponders (66).

Gut microbiome is also associated with toxicity, as supported by an analysis involving 77 patients with advanced melanoma treated with anti-CTLA-4 in combination with anti-PD-1 therapy (66). Moreover, fecal material transplantation may modulate the response to ICI. Preclinical studies have demonstrated that when germ-free mice are treated with fecal microbiome transplants from ICI responders, these mice also respond to ICI therapy. In contrast, the mice did not respond to ICI therapy when the stool material was from patients who did not respond to ICI (67). A Phase I clinical trial evaluating the safety and feasibility of fecal material transplantation in 10 patients with anti-PD-1 refractory metastatic melanoma successfully induced 1 complete response and 2 partial responses (68). The mechanisms underlying the influence of gut microbiota on the efficacy and toxicity of immunotherapy remain to be further demonstrated.

### 3.4 Pregnancy

Pregnancy can be divided into three trimesters, and the immune state also undergoes three phases. First, there is a pro-inflammatory phase in the first trimester, during which the embryo is implanted and the placenta is formed (69). Second, an anti-inflammatory phase in the second and third trimesters is necessary for fetal tolerance (70). Lastly, the immune state switches back to a pro-inflammatory phase during delivery for uterine contraction and placental expulsion (69).

The local immune status at the mother-fetus interface, or by another name, uterine decidua, is critical for fetal-maternal tolerance. The uterine decidua consists of trophoblasts, decidual stromal cells, and immune cells (71). Throughout pregnancy, the fluctuations and interactions of these cells aid in trophoblast invasion and protect the fetus from rejection by the mother (71). Here we focus on features that are directly related to common immunotherapies. PD-1 and PD-L1 form a co-inhibitory signal that modulates T cell activation and is important for fetal-maternal tolerance (72). PD-1 is primarily expressed by lymphocytes, with levels increasing in deciduous lymphocytes and decreasing in peripheral lymphocytes during the first trimester (72). PD-L1 is expressed by decidual stromal cells and trophoblasts, and the expression levels of PD-L1 increases from the 10-12 weeks after implantation (73). CTLA-4 and CD80/86 are also important inhibitory signals. CTLA-4 is predominantly expressed on Tregs, which show a constant expression during pregnancy (74). CD80/86 are costimulatory molecules on decidual stromal cells, and may contribute to the Th2 propensity of decidual DCs (75). Common ICI drugs target both signaling pathways. Therefore, when pregnant women require immunotherapy, the pregnancy may be disrupted and fluctuations in the expression of target molecules may affect immunotherapy.

Immunotherapy is rarely administered during pregnancy because of concerns about the potential effects on the fetus. For ICIs, seven cases of women becoming pregnant while undergoing ICI treatment have been reported (76–81), and four cases of ICI therapy beginning during pregnancy, with or without chemotherapy (76, 82–84). Three of the melanoma mothers showed disease progression after delivery (80, 82, 83), and one had an emergency Caesarean section at 24 weeks gestation due to tumor progression and died the day after surgery (82). Five placentas were pathologically examined in these studies, including one from a patient with metastatic melanoma that showed several metastases on the maternal side (82). During follow-up, none of the children showed signs of tumor metastasis. Three women developed irAE, one with diarrhea (83) and two with hepatotoxicity (79), and one of the latter discontinued ICIs (80). A case report indicated that exposure to ICIs may cause irAE in newborns (82). For therapeutic vaccines, Calmette-Guerin (BCG) is the tuberculosis vaccine and could be used to treat bladder cancer by injecting it into the bladder. A female was diagnosed with bladder cancer at 36 weeks gestation and treated with BCG. She gave birth to a healthy baby and continued breast-feeding after the baby received the intradermal BCG vaccine (85).

### 3.5 Virus infection

Approximately 10-12% of all newly diagnosed cancer cases worldwide are associated with viral infections (86). Eight viruses have been found to contribute to cancer development, including human papilloma virus(HPV), hepatitis B and C virus (HBV/HCV) (86). Viruses affect host immunity and cell malignancy through several mechanisms, such as directly increasing genomic instability promoting tumor cells (87), indirectly providing an environment for tumor progression by inducing chronic inflammation (88), and impairing the immune system preventing tumor cells from being excluded (89). Varies immunotherapy methods have been developed based on the association of the virus and the pathological process of cancer. Certain viral proteins are continuously expressed in tumor cells, and tumor cells may be specifically killed by targeting these proteins, known as therapeutic vaccines (90). Several therapeutic vaccines have entered clinical trials (90). Another approach is infusion of T cells carrying native TCR, known as ACT therapy (91). However, whether viral infection status in tumor patients affect immunotherapy has not been fully elucidated.

#### 3.5.1 SARS-CoV-2

The COVID-19 pandemic has and will inevitably have a long-term impact on world health, and it is of interest to see how SARS-CoV-2 infection in cancer patients affects immune status and immunotherapy. SARS-CoV-2 is a single-stranded RNA virus. Its spike protein interacts with ACE2 to facilitate cellular invasion by the virus and stimulate immunity. In the first few days after SARS-CoV-2 infection, innate immune cells identify pathogen associated molecular pattern or damage associated molecular pattern via pattern recognition receptors (PRRs). These PRRs are triggered, causing a substantial release of cytokines that exert direct antiviral effects and activate downstream immune responses (92). The severity of COVID-19 is associated with immune imbalance and sustained release of high levels of cytokines, not viral load (93). Cancer patients are often accompanied by immune balances, weakened immune cells, and destruction of immune-related anatomical structures, making them more vulnerable to SARS-CoV-2 infections. Tumor type, active tumor, and advanced tumor stage are risk factors for death from COVID-19 (94).

SARS-CoV-2 infection can cause long-term perturbations in immune status (95). Notably, change of immune cells, antibody production, and cytokine release due to SARS-CoV-2 infection are influenced by confounding factors such as age, gender, and tumor treatment, which should be taken into consideration comprehensively (96). Tumors and COVID-19 share similar immune processes, such as excessive cytokine release and weakened humoral and cellular immunity. Immunotherapy can elevate IFN-γ expression, thereby increasing ACE2 expression which makes patients receiving immunotherapy more susceptible to SARS-CoV-2 infection (97). This evidence suggests the complexity of the immune status in the coexistence of tumors and SARS-CoV-2, presenting a challenge for immunotherapy and ID.

Vaccination against SARS-CoV-2 is one of the most critical measures to reduce COVID-19 mortality. However, the vaccination efficacy (VE) for tumor patients (62-72%) is lower than that of the normal population (94%), with hematologic tumors demonstrating even lower VE compared to solid tumors (98). The treatment of tumors with chemotherapy, anti-CD20, anti-CD38, and CAR-T has been found to disrupt the humoral immune response induced by SARS-CoV-2 vaccine and impair the VE, while surgery, ICIs, endocrine therapy and radiotherapy did not affect the VE (98, 99). In addition, tumor patients often experience long-term chronic depletion, requiring repeated consolidation of immune memory. A study found that administering a third dose of the SARS-CoV-2 vaccine increased the detection rate of Omicron-specific serum antibodies in tumor patients from 47.8% to 88.9% (100). COVID-19 should be considered a long-term infection and be included in the ID model. Treatment decisions should be based on comprehensive assessment of patients’ multiple diseases.

The safety and prognosis of comorbidities with cancer immunotherapy and SARS-CoV-2 infection remain controversial. Several studies have indicated that ICIs may increase the risk of COVID-19-related deaths (101). The mechanism behind this involves over-activation of CD8+ T cells, which not only promotes acute respiratory diseases, but also causes subsequent suppresses of cellular immunity, providing an opportunity for tumor cells to thrive. Severe symptoms and need for hospitalization due to SARS-CoV-2 infection have been reported in 39 - 54% of cancer patients, a higher rate compared to individuals without tumors (102). However, other studies have contradicted these findings, demonstrating that ICIs do not increase mortality due to COVID-19, and can even enhance the immune system’s specific response to the virus, which is associated with developed OS (103, 104). These conflicting results may be related to disease type, cancer stage, and immune system status, highlighting the importance of ID.

There are certain commonalities between cancer immunotherapy adverse events and the pathogenesis of COVID-19. For example, the co-occurrence of pulmonary irAEs and COVID-19 pneumonia increases the potential risk of interstitial inflammatory infiltration and diffuse alveolar damage, thereby increasing the likelihood of death from terminal respiratory failure (105). Additionally, there are similarities between the process of acute respiratory distress syndrome caused by SARS-CoV-2 through cytokine storm and cytokine release syndrome after CAR-T treatment (106, 107). Cancer patients treated with CD19 CAR-T therapy may develop B cell aplasia, which impairs the antiviral humoral immune response and puts them at increased risk for complications of SARS-CoV-2 infection (97). In conclusion, there are many interactions of the pathological processes and immune mechanisms between viral infections and cancer. Therefore, the predictive value of including viruses in ID models for cancer and immunotherapy should be appreciated.

#### 3.5.2 HBV/HCV

Recent evidence suggests that HBV/HCV may affect cancer immunotherapy. A multicenter retrospective cohort of 180 patients with advanced CRC treated with anti-PD-1 found that HBV patients had higher mismatch repair defects and fewer cancer metastases than non-HBV patients (108). Nevertheless, there was no statically significant difference in the ORR (both of 39%) between HBV and non-HBV group. Notably, the CR rate in the HBV group (17 CR, 13 PR) was higher than in the non-HBV group (11CR, 19PR). Whether this indicates that HBV infection favors anti-PD-1 therapy remains to be further investigated. Another retrospective study, which included 50 cancer patients with HIV and/or HBV/HCV infection, found no significant association between viral load and anti-tumor immune response (109).

#### 3.5.3 HIV

HIV can damage human T cells and cause acquired immunodeficiency syndrome (AIDS), and there are approximately 3782700 HIV-infected individuals worldwide. However, people with HIV are generally excluded from immunotherapy cohorts, and most studies of HIV and cancer treatment have been conducted in Europe and the United States, rather than in Asia, Africa and Latin America, where 75 percent of HIV patients live (110). Therefore, few clinical trials have provided guidance for personalized treatment of HIV in cancer patients. A phase I clinical trial found that Pembrolizumab is safe for the treatment of advanced cancer in HIV-infected patients with a CD4+ T cell count of greater than 100 cells/μL (111). To recap, all of these evidence supports the inclusion of more virus-infected cancer patients for immunotherapy in the future to further determine the impact of infection on cancer immunotherapy, thereby developing the ID model with the concern of virus infection.

## 4 Biomarkers: direct predictive factors in ID

The outcome of immunotherapy is highly heterogeneous among individuals. Early practice has demonstrated that when specific therapies are used to treat specific diseases, there are biomarkers that may partially fulfill the function of ID as envisioned. To enumerate all biomarkers and describe them in detail is not the focus of this paper. Instead, we would like to try to discuss the characteristics of ideal biomarkers to provide a reference for the construction of ID systems. Moreover, we will provide some successful cases to illustrate the feasibility of this idea.

An ideal biomarker should be accurate, discriminative between the population of interest and controls, and repeatable. Biomarkers from peripheral blood are an attractive option because they are relatively non-invasive and can be taken multiple times. Biomarkers within imaging methods such as CT/MRI are also worth investigating. Biomarkers should be involved in pathogenesis mechanisms and related to disease activity or therapeutic targets.

Tumor-related biomarkers have been widely discussed. We analyze the role and characteristics of the major biomarkers in the tumor-immune interaction mechanism and summarize these biomarkers into five categories.

Some biomarkers reflect the tumor’s ability to stimulate the immune system. Deficient mismatch repair (dMMR) means the loss of expression of mismatch repair proteins that could correct mismatched bases during DNA replication, so the DNA replication errors at microsatellite regions accumulated, causing microsatellite instability-high (MSI-H). Furthermore, patients with MSI-H/dMMR may have more tumor associated antigens (TAAs) and tumor specific antigens (TSAs) that could stimulate the immune system. The tumor mutation burden (TMB) is the genetic mutation rate of tumor cells, and is also associated with the TSAs, also known as neoantigens. The neoantigenic burden is dominated by TSAs targeted by T cells. Recent studies have provided evidence that MSI-H/dMMR (112), TMB (113), and neoantigen burden (114) are emerging as promising biomarkers for clinical outcomes in cancer immunotherapy. Also, Marta and colleagues build a neoantigen fitness model based on immune interactions of neoantigens that could predict survival in melanoma patients and lung cancer patients treated with ICIs. These studies demonstrate the potential of neoantigens and related gene backgrounds as ID models, and suggest that ID may reveal new therapeutic targets.

Another dimension is the immune system’s ability to recognize malignant cells. CD8+ T cell dependent killing of cancer cells requires appropriate presentation of tumor antigens by MHC, which in humans is human leukocyte antigen (HLA) molecules, resulting in at least three kinds of biomarkers: specific HLA genotype for certain cancer type (115), some kind of HLA alleles having strong antigen presentation ability (116), and high HLA diversity which could provide a large library and are more likely to have appropriate HLA (117). An exploratory study of multiple myeloma patients treated with bortezomib found some HLA alleles as candidates, as patients carrying HLA-DQB1*03:02, HLA-DQB1*05:01, and HLA-DRB1*01:01 class II alleles are more likely to get a complete response (115). In 1535 advanced cancer patients treated with ICIs, the HLA-B44 supertype is associated with extended survival, whereas the HLA-B62 supertype was associated with poor outcomes (116). In patients with kidney cancer treated with Lenvatinib and Pembrolizumab, it has been found that HLA-I evolutionary divergence is associated with both improved clinical benefit and response durability (117).

Tumors exploit multiple mechanisms to evade immune recognition, and several features associated with immune evasion could be excellent predictors. Overexpression of the PD-L1 protein (a kind of immune checkpoint) on the cancer cells is a major immune evasion mechanism, and antibodies to blockade the PD-1/PD-L1 interaction could normalize anti-tumor immunity. PD-L1 expression levels are the first and most investigated biomarkers to predict prognosis with respect to ICIs for certain cancer types. KEYNOTE-024 provided the highest level of clinical evidence certifying that immunotherapy accompanied with PD-L1 diagnosis could bring nearly clinical cure outcome to advanced non-small cell lung cancer (NSCLC) (118). For solid tumors, CD8+ T cells need to infiltrate into the tumor to contact cancer cells and kill them, but the TME may exclude T cells (119). Levels of intratumoral tumor infiltrating lymphocytes were associated with a better prognosis in epithelial ovarian cancer (120). TME is rich in immunosuppressive cytokines and cells, and may cause T cell depletion and inhibit anti-tumor immunity. Researchers had analyzed the immune cell composition and transcriptomic features of hepatocellular carcinoma samples, and defined a 9-gene signature related to T cell exhaustion, whose expression was higher in responders, and independently predicted better progression free survival (PFS) and overall survival (OS) (121).

Some markers can reflect the actual immune system response to the tumor. For example, peripheral tumor antigen-specific T cell expansion suggests a large therapeutic response. A clinical trial of patients with metastatic urothelial carcinoma treated with anti-PD-L1 demonstrated a higher number of neoantigen-specific CD8+ T cells in the peripheral blood compared to disease progression in patients with control disease (122). Other studies suggest the peripheral blood neutrophil to lymphocyte ratio (NLR) as a negative prognosis predictor of immunotherapy. A phase III trial of advanced gastric cancer patients treated with nivolumab showed that low blood NLR (≤2.9, median) was associated with better PFS and OS (123), as lower blood NLR reflect to higher lymphocytes expansion after immunotherapy.

With the development of sequencing and bioinformatics, a growing number of studies have identified a number of genomic, transcriptomic, or protein signatures associated with immunotherapy outcomes. However, it remains unclear what is the underlying mechanism behind these features affecting immunotherapy. Numerous studies have constructed predictive models by mining public or private databases. For instance, Qing Liu and colleagues screened 1018 differentially expressed immunologic genes (DEGs) of a dataset consisting of 414 bladder cancer samples and 19 normal samples from The Cancer Genome Atlas (TCGA), and constructed a predict risk model consisting of 15 genes (124). They validated the model in another dataset consisting of RNA-seq data from 48 tumor tissues and the relevant clinical information, GSE19423, from the Gene Expression Omnibus (GEO). The proposed model demonstrated good predictive power in predicting OS risk in the validation dataset. They reviewed the literature and found that 10 out of 15 genes are involved in TME, with the mechanism still to be investigated further. Similar studies have sprung up in recent years, but are still far from clinical practice. This type of research is promising as a prototype for an ideal ID system with validation in larger external datasets, including more dimensional variables, combined with a deeper understanding of immune mechanisms.

## 5 AI helps to construct ID system

**Figure 2:**
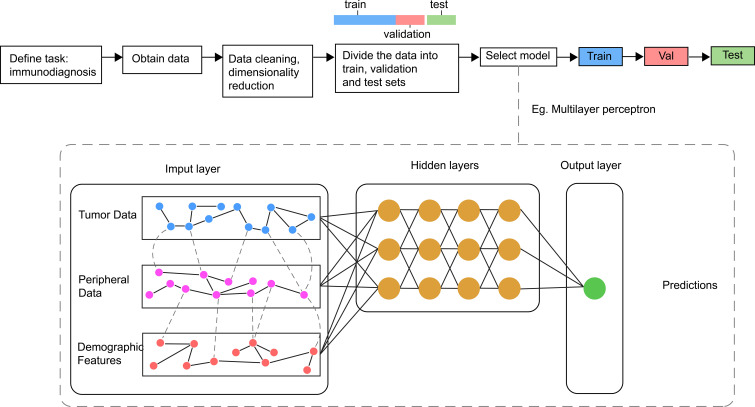
Main processes and sample model of AI in Immunodiagnosis. Different data related with immune status are collected and processed before inputted into the model for the training, validating and testing. In general, the model consists of one input layer, many hidden layers and one output layer. The output layer is associated with different labels refer to outcomes of immunotherapy.

AI refers to the use of machines to imitate intelligent behavior for performing complex tasks with minimal human intervention. Machine Learning (ML) is a branch of AI, which involved the use of algorithms such as Logistic Regression, Decision Trees, Random Forests, and Support Vector Machines. Deep Learning (DL) and Artificial Neural Networks represent new frontiers in ML that encompass Convolutional Neural Networks (CNN) and Recurrent Neural Networks (RNN). CNN offer unique advantages for image processing applications and have been successfully employed for feature extraction in clinical imaging data. RNN is often used for the analysis of time-series data and has shown advantages in dynamic monitoring of disease. Additionally, DL can directly process unstructured data such as images, sounds, and languages, making it particularly suitable for clinical medical record texts, image classification, and tumor diagnosis and treatment (125).The main processes of AI are shown in **Figure 2**.

AI has been applied to multiple medicine fields such as diabetes (126), including artificial pancreas (calculate and inject insulin dosage automatically) and continuous blood glucose prediction; ophthalmology (127), including detecting diabetic retinopathy and macular oedema. In recent years, significant progress has been made in the research of AI application for early tumor diagnosis. Studies have demonstrated that AI can achieve comparable accuracy and specificity to specialist physicians in diagnosing various cancers, such as breast cancer (128), lung cancer (129), skin tumors (130), and ovarian cancer (131). In addition to accurate identification and early diagnosis of cancer, AI can also assist in long-term follow-up and health management of cancer recurrence (132).

ID is a challenging prediction problem. The input dataset should be large enough and contain enough representative features. An ideal ID system requires simple, inexpensive, and reproducible detection techniques. The rapid development of microfluidic chip platforms in recent years has provided a miniaturized and highly controlled environment for the occurrence of biochemical reactions. It is also compatible with analytical methods, and can give rapid detection results from trace samples (133). Another area that has received a lot of attention is wearable devices. Wearable devices can collect health information noninvasively and continuously, and have shown promising potential to support and implement medical decisions (134). These innovations in detection and monitoring methods, combined with AI, promise to expand the dataset amount.

### 5.1 Opportunities of AI in precise immunotherapy

#### 5.1.1 Standardizing the diagnostic criteria for existing biomarkers

Currently, immunohistochemistry (IHC) detection of PD-L1 expression as a predictive biomarker for ICIs has been clinically implemented. The staining results are semi-quantitatively evaluated by pathologists. However, due to the heterogeneous expression of PD-L1 in tumor cells and various immune cells, manual interpretation lacks consistency and reproducibility. Moreover, accurate expression values cannot be provided, and manual scoring is subjective, leading to diagnostic bias. To address this issue, several studies have utilized AI for quantitative analysis of digital slides. The established models demonstrated good consistency with human experts’ scores and have significantly improved the diagnostic efficiency of untrained pathologists (135–137).

#### 5.1.2 Identify unstructured data

Traditional statistical methods are often insufficient to extract features from high-dimensional clinical images such as CT, MRI, and PET/CT, while subjective interpretation by clinical experts can lead to bias. Recently, advances in AI-based medical image biomarkers have shown great potential for noninvasive characterization of tumors and TME, enabling patient selection and efficacy prediction for immunotherapy. For instance, AI has been utilized to automatically analyze CT features of NSCLC and melanoma patients, resulting in the development of a noninvasive radiomic biomarker that effectively distinguished immunotherapy responders and non-responders (138). Odors are also unstructured data. Using AI technology, researchers have trained a device called the “electronic nose” to detect volatile organic compound patterns in exhaled breath that were related to the response of NSCLC patients to anti-PD-1, enabling accurate prediction of treatment outcomes (139).

#### 5.1.3 Developing personalized drugs

The combination of AI and multi-omics data holds the potential of developing personalized drugs quickly. Researchers have developed an AI-based platform named PIONER for target discovery that enabled the selection of neoantigens suitable for personalized DNA vaccine EVX-02. The approach involved sequencing both tumor and healthy tissues from cancer patients, identifying genetic mutations in the tumor tissue through comparison with healthy tissue, and utilizing AI to predict which mutations are most likely to generate neoantigens capable of elicitin an immune response in patients. The I/IIa clinical trial of EVX-02 combined with nivolumab achieved good results, with no instances of recurrence observed among the 10 patients enrolled during the trial period (140).

#### 5.1.4 Integrated multidimensional unstructured data to build ID model

The complexity of tumor-immune interactions necessitates a multi-dimensional model for accurate prediction. To this end, Timothy Chan’s team has comprehensively integrated multiple biological features relevant to immunotherapy efficacy, including but not limited to TMB, MSI, BMI, sex, NLR, tumor stage/type, and age. They included 1,479 patients across 16 cancer types and established two AI models named RF11 and RF16 that incorporated 11 and 16 biological features, respectively. In the training set, RF16 had an AUC exceeding 0.8 in various cancers, far surpassing the independent predictive efficacy of single indicator (~0.6) (141). Another study found that merely measuring the quantity of TILs cannot accurately reflect the tumor-immune interactions and the functional status of T cells and developed an AI-based PhenoTIL system incorporating multidimensional factors. The PhenoTIL system exhibited a superior AUC (0.738 versus 0.683) compared to TNM staging in NSCLC patients (142).

#### 5.1.5 An ID system in the whole process of tumor diagnosis, treatment, and follow-up

The explosive growth of biology data and the development of portable devices to monitor patients’ health state enable the application of AI on generating tumor decision support ID systems. AI can be used to optimize immunotherapy methods in search of a balance between efficacy, adverse reactions, and cost (143). AI could also be used to predict the risk of recurrence. Patients with low recurrence risk can avoid unnecessary radiation exposure and tedious hospital follow-ups, improving their quality of life (132). These findings, along with numerous emerging findings, strongly support the use of AI in facilitating precision immunotherapy.

### 5.2 Barriers to adopting AI in the clinical transformation

Despite the notable advancements in immune evaluation facilitated by AI, the clinical transformation practice of this technology remains confronted with several challenges that can be categorized into three distinct aspects:

#### 5.2.1 Accessibility of big data

The efficacy of AI is optimized when it is trained and validated on extensive data sets. However, the paucity of publicly available data may be attributed to the imperative of safeguarding patient privacy or commercial conflicts of interest. Consequently, it is imperative to establish a comprehensive public data platform of considerable magnitude. Zlatko and colleagues have created The Cancer Immunome Atlas (https://tcia.at/) to characterize the intratumoral immune landscapes of 20 solid cancers and used machine learning to develop a scoring scheme for the quantification termed immunophenoscore, which showed the predicted value of response to CTLA-4 and PD-1 inhibitors in two independent cohorts (144). In addition, medical records consist of a variety of unstructured data types, including text, images, and voice. In order to enable effective input of this information for use by AI, it is necessary to create a uniform data format.

#### 5.2.2 Open the black box

The nature of AI algorithms is often referred to as ‘black box’, the output of which is difficult to interpret for the engineers who develop it and for the doctors and patients who use it. Laboratory studies may be required to provide a biological basis, but it may take more time.

#### 5.2.3 What if AI made a mistake?

In situations where AI produces errors, it is essential to determine how to identify and address these inaccuracies. Furthermore, if a mistake made by AI impairs the health or well-being of a patient, it becomes necessary to assign responsibility for the error. A study involving 657 NSCLC patients entered 34 clinical data into an AI model and compared the combination of 8 feature reduction methods and 10 machine learning classification algorithm (145). The researchers discovered notable variances in the AUC among multiple combinations, and the best combinations for predicting RFS, recurrence, and death were different, which suggested us to select appropriate AI approaches for different clinical scenarios. One plausible solution is to incorporate multiple AI algorithms and feature selection methods concurrently. Additionally, a group of human experts should review when different AI models yield conflicting outcomes.

**Figure 3:**
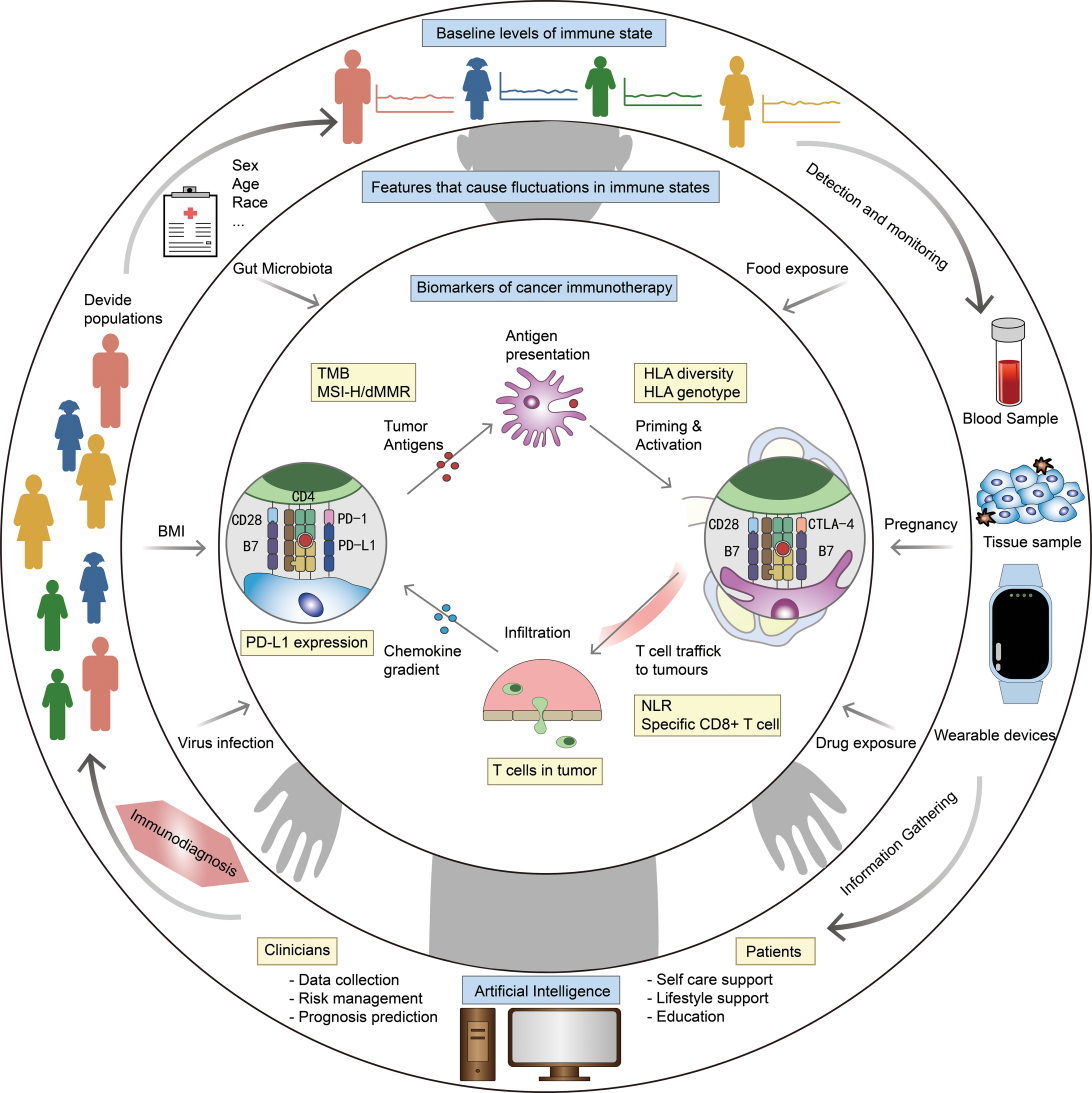
Blueprint of ID model and ID system. Outer ring: factors related to baseline level of immune status, eg. sex and age. Middle ring: factors that cause fluctuations in immune status, eg. body mass index (BMI) and gut microbiota. Inner ring: biomarkers for specific diseases and therapies, eg. PD-L1 expression and HLA diversity. The outer ring describes our vision for ID system: stratify patients based on medical record information, get more information from blood/tissue samples and wearable devices, integrate all information by AI to make ID, and optimize the diagnostic model at any time based on clinical feedback.

We envisioned the components of an ID model and a blueprint for using AI to establish an ID system for cancer management (**Figure 3**). The model can diagnose the immune status of patients, determine whether they are suitable for immunotherapy, and even recommend best therapeutic strategies. From the review above, we summarized that the model contains three levels of features: demographic characteristics obtained from the patients’ medical record to determine a baseline immune level; some variable physiological or pathological factor, reflects the influence of the patient’s current health level on immune status; cellular, molecular and genetic characteristics obtained from the patients’ tumor histopathologic and blood samples, serve as biomarkers that match the tumor types and therapeutic strategies. By making immunotherapy decision with ID model, and continuously evaluating patients’ immune status and immune response through wearable devices and other monitoring methods, it is expected to contribute to the precision of tumor immunotherapy.

## 6 Conclusion

In this paper, we first present the important concept of ID and describe the method to construct an ID system. There is significant individual heterogeneity in the outcomes of immunotherapy for immune-related diseases and ID should be performed prior to treatment planning. Different demographic characteristics, physiological and pathological conditions have many disturbing effects on the human immune system. As a result, management protocols for patients should be tailored to address their needs at different points in the course of the disease. Depending on the treatment mechanism, there may be some characteristic biomarkers. Combining cutting-edge AI methods to integrate multidimensional information will hopefully build an ideal ID system. Of course, the blueprint of ID system we came up with is just a prototype of an ideal system. With the in-depth research on the mechanism of immune and tumor development and immunotherapy, as well as the continuous iteration of AI, we can expect more accurate and sensitive ID system to be applied to real clinical practice.

## Author contributions

RW and KX wrote the initial draft and draw the figures for clear presentation. ZW had a critically overview of the entire manuscript and substantially revised it. DW had a critically overview of the entire manuscript and provided expertise on the machine learning. BH complemented the references and optimized the figures for better presentation. JR and CS provided professional advice on artificial intelligence and complemented cutting-edge research. DM had an editorial overview of the entire manuscript and supervised the whole program. SL and LL did the seminar design and provided scientific expertise, guidance and had and editorial overview of the entire manuscript. All authors contributed to the article and approved the submitted version.

## References

[1] Parkin J Cohen B. An overview of the immune system. Lancet (2001) 357(9270):1777–89. doi: 10.1016/S0140-6736(00)04904-7 11403834

[2] Zsiros E Lynam S Attwood KM Wang C Chilakapati S Gomez EC. Efficacy and safety of pembrolizumab in combination with bevacizumab and oral metronomic cyclophosphamide in the treatment of recurrent ovarian cancer: a phase 2 nonrandomized clinical trial. JAMA Oncol (2021) 7(1):78–85. doi: 10.1001/jamaoncol.2020.5945 33211063 PMC7677872

[3] Reckamp KL Redman MW Dragnev KH Minichiello K Villaruz LC Faller B. Phase II randomized study of ramucirumab and pembrolizumab versus standard of care in advanced non-Small-Cell lung cancer previously treated with immunotherapy-Lung-MAP S1800A. J Clin Oncol (2022) 40(21):2295–306. doi: 10.1200/JCO.22.00912 PMC9287284 35658002

[4] Haslam A Prasad V. Estimation of the percentage of US patients with cancer who are eligible for and respond to checkpoint inhibitor immunotherapy drugs. JAMA Netw Open (2019) 2(5):e192535. doi: 10.1001/jamanetworkopen.2019.2535 31050774 PMC6503493

[5] Sullivan RJ Weber JS. Immune-related toxicities of checkpoint inhibitors: mechanisms and mitigation strategies. Nat Rev Drug Discovery (2022) 21(7):495–508. doi: 10.1038/s41573-021-00259-5 34316029

[6] Shen-Orr SS Furman D Kidd BA Hadad F Lovelace P Huang YW. Defective signaling in the JAK-STAT pathway tracks with chronic inflammation and cardiovascular risk in aging humans. Cell Syst (2016) 3(4):374–384.e4. doi: 10.1016/j.cels.2016.09.009 27746093 PMC5358544

[7] Carr EJ Dooley J Garcia-Perez JE Lagou V Lee JC Wouters C. The cellular composition of the human immune system is shaped by age and cohabitation. Nat Immunol (2016) 17(4):461–8. doi: 10.1038/ni.3371 PMC4890679 26878114

[8] Di Florio DN Sin J Coronado MJ Atwal PS Fairweather D. Sex differences in inflammation, redox biology, mitochondria and autoimmunity. Redox Biol (2020) 31:101482. doi: 10.1016/j.redox.2020.101482 32197947 PMC7212489

[9] Gautier T Ziegler LB Gerber MS Campos-Náñez E Patek SD. Artificial intelligence and diabetes technology: a review. Metabolism (2021) 124:154872. doi: 10.1016/j.metabol.2021.154872 34480920

[10] Elemento O Leslie C Lundin J Tourassi G. Artificial intelligence in cancer research, diagnosis and therapy. Nat Rev Canc (2021) 21(12):747–52. doi: 10.1038/s41568-021-00399-1 34535775

[11] Wang L Wang FS Gershwin ME. Human autoimmune diseases: a comprehensive update. J Intern Med (2015) 278(4):369–95. doi: 10.1111/joim.12395 26212387

[12] Haupt S Caramia F Klein SL Rubin JB Haupt Y. Sex disparities matter in cancer development and therapy. Nat Rev Cancer (2021) 21(6):393–407. doi: 10.1038/s41568-021-00348-y 33879867 PMC8284191

[13] Lakshmikanth T Muhammad SA Olin A Chen Y Mikes J Fagerberg L. Human immune system variation during 1 year. Cell Rep (2020) 32(3):107923. doi: 10.1016/j.celrep.2020.107923 32697987

[14] Huang Z Chen B Liu X Li H Xie L Gao Y. Effects of sex and aging on the immune cell landscape as assessed by single-cell transcriptomic analysis. Proc Natl Acad Sci U.S.A. (2021) 118(33):e2023216118. doi: 10.1073/pnas.2023216118 34385315 PMC8379935

[15] Conforti F Pala L Bagnardi V De Pas T Martinetti M Viale G. Cancer immunotherapy efficacy and patients’ sex: a systematic review and meta-analysis. Lancet Oncol (2018) 19(6):737–46. doi: 10.1016/S1470-2045(18)30261-4 29778737

[16] Ye Y Jing Y Li L Mills GB Diao L Liu H. Sex-associated molecular differences for cancer immunotherapy. Nat Commun (2020) 11(1):1779. doi: 10.1038/s41467-020-15679-x 32286310 PMC7156379

[17] Unger JM Vaidya R Albain KS LeBlanc M Minasian LM Gotay CC. Sex differences in risk of severe adverse events in patients receiving immunotherapy, targeted therapy, or chemotherapy in cancer clinical trials. J Clin Oncol (2022) 40(13):1474–86. doi: 10.1200/JCO.21.02377 PMC9061143 35119908

[18] Thakkar JP Villano JL McCarthy BJ. Age-specific cancer incidence rates increase through the oldest age groups. Am J Med Sci (2014) 348(1):65–70. doi: 10.1097/MAJ.0000000000000281 24805784 PMC4119927

[19] Park H Bourla AB Kastner DL Colbert RA Siegel RM. Lighting the fires within: the cell biology of autoinflammatory diseases. Nat Rev Immunol (2012) 12(8):570–80. doi: 10.1038/nri3261 PMC4165575 22828911

[20] Nikolich-Žugich J. The twilight of immunity: emerging concepts in aging of the immune system. Nat Immunol (2018) 19(1):10–9. doi: 10.1038/s41590-017-0006-x 29242543

[21] Berben L Floris G Kenis C Dalmasso B Smeets A Vos H. Age-related remodelling of the blood immunological portrait and the local tumor immune response in patients with luminal breast cancer. Clin Transl Immunol (2020) 9(10):e1184. doi: 10.1002/cti2.1184 PMC7532981 33024560

[22] Hu W Liu H Li Z Liu J Chen L. Impact of molecular and clinical variables on survival outcome with immunotherapy for glioblastoma patients: a systematic review and meta-analysis. CNS Neurosci Ther (2022) 28(10):1476–91. doi: 10.1111/cns.13915 PMC9437230 35822692

[23] Rittmeyer A Barlesi F Waterkamp D Park K Ciardiello F von Pawel J. Atezolizumab versus docetaxel in patients with previously treated non-small-cell lung cancer (OAK): a phase 3, open-label, multicentre randomised controlled trial. Lancet (2017) 389(10066):255–65. doi: 10.1016/S0140-6736(16)32517-X PMC6886121 27979383

[24] Yau T Hsu C Kim TY Choo SP Kang YK Hou MM. Nivolumab in advanced hepatocellular carcinoma: sorafenib-experienced Asian cohort analysis. J Hepatol (2019) 71(3):543–52. doi: 10.1016/j.jhep.2019.05.014 31176752

[25] Finn RS Ryoo BY Merle P Kudo M Bouattour M Lim HY. Pembrolizumab as second-line therapy in patients with advanced hepatocellular carcinoma in KEYNOTE-240: a randomized, double-blind, phase III trial. J Clin Oncol (2020) 38(3):193–202. doi: 10.1200/JCO.19.01307 31790344

[26] Spigel DR McCleod M Jotte RM Einhorn L Horn L Waterhouse DM. Safety, efficacy, and patient-reported health-related quality of life and symptom burden with nivolumab in patients with advanced non–small cell lung cancer, including patients aged 70 years or older or with poor performance status (CheckMate 153). J Thorac Oncol (2019) 14(9):1628–39. doi: 10.1016/j.jtho.2019.05.010 31121324

[27] Kugel CH III Douglass SM Webster MR Kaur A Liu Q Yin X. Age correlates with response to anti-PD1, reflecting age-related differences in intratumoral effector and regulatory T-cell populations. Clin Cancer Res (2018) 24(21):5347–56. doi: 10.1158/1078-0432.CCR-18-1116 PMC6324578 29898988

[28] Betof AS Nipp RD Giobbie-Hurder A Johnpulle RAN Rubin K Rubinstein SM. Impact of age on outcomes with immunotherapy for patients with melanoma. Oncologist (2017) 22(8):963–71. doi: 10.1634/theoncologist.2016-0450 PMC5553960 28476944

[29] Pasquini MC Locke FL Herrera AF Siddiqi T Ghobadi A Komanduri KV. Post-marketing use outcomes of an anti-CD19 chimeric antigen receptor (CAR) T cell therapy, axicabtagene ciloleucel (Axi-cel), for the treatment of Large b cell lymphoma (LBCL) in the united states (US). Blood (2019) 134:764. doi: 10.1182/blood-2019-124750

[30] Motzer RJ Tannir NM McDermott DF Arén Frontera O Melichar B Choueiri TK. Nivolumab plus ipilimumab versus sunitinib in advanced renal-cell carcinoma. N Engl J Med (2018) 378(14):1277–90. doi: 10.1056/NEJMoa1712126 PMC5972549 29562145

[31] Siegel RL Miller KD Fuchs HE Jemal A. Cancer statistics, 2022. CA Cancer J Clin (2022) 72(1):7–33. doi: 10.3322/caac.21708 35020204

[32] Zhang C Zhang C Wang Q Li Z Lin J Wang H. Differences in stage of cancer at diagnosis, treatment, and survival by race and ethnicity among leading cancer types. JAMA Netw Open (2020) 3(4):e202950. doi: 10.1001/jamanetworkopen.2020.2950 32267515 PMC7142383

[33] Chng WJ Tan GB Kuperan P. Establishment of adult peripheral blood lymphocyte subset reference range for an Asian population by single-platform flow cytometry: influence of age, sex, and race and comparison with other published studies. Clin Diagn Lab Immunol (2004) 11(1):168–73. doi: 10.1128/CDLI.11.1.168-173.2004 PMC321350 14715565

[34] Cairo C Armstrong CL Cummings JS Deetz CO Tan M Lu C. Impact of age, gender, and race on circulating γδ T cells. Hum Immunol (2010) 71(10):968–75. doi: 10.1016/j.humimm.2010.06.014 PMC2941533 20600446

[35] Brunner PM Guttman-Yassky E. Racial differences in atopic dermatitis. Ann Allergy Asthma Immunol (2019) 122(5):449–55. doi: 10.1016/j.anai.2018.11.015 30465859

[36] Longo DM Louie B Mathi K Pos Z Wang E Hawtin RE. Racial differences in b cell receptor signaling pathway activation. J Transl Med (2012) 10:113. doi: 10.1186/1479-5876-10-113 22672557 PMC3464787

[37] Printz C. Neurotoxicity more likely in Hispanic children treated for acute lymphoblastic leukemia. Cancer (2019) 125(4):494–5. doi: 10.1002/cncr.31989 30698835

[38] Bai X Shoushtari AN Betof Warner A Si L Tang B Cui C. Benefit and toxicity of programmed death-1 blockade vary by ethnicity in patients with advanced melanoma: an international multicentre observational study. Br J Dermatol (2022) 187(3):401–10. doi: 10.1111/bjd.21241 35293617

[39] Ayers KL Mullaney T Zhou X Liu JJ Lee K Ma M. Analysis of real-world data to investigate the impact of race and ethnicity on response to programmed cell death-1 and programmed cell death-ligand 1 inhibitors in advanced non-small cell lung cancers. Oncologist (2021) 26(7):e1226–39. doi: 10.1002/onco.13780 PMC8265370 33829580

[40] Faruqi AJ Ligon JA Borgman P Steinberg SM Foley T Little L. The impact of race, ethnicity, and obesity on CAR T-cell therapy outcomes. Blood Adv (2022) 6(23):6040–50. doi: 10.1182/bloodadvances.2022007676 PMC9700270 35939781

[41] Jaratlerdsiri W Chan EKF Gong T Petersen DC Kalsbeek AMF Venter PA. Whole-genome sequencing reveals elevated tumor mutational burden and initiating driver mutations in African men with treatment-naïve, high-risk prostate cancer. Cancer Res (2018) 78(24):6736–46. doi: 10.1158/0008-5472.CAN-18-0254 30217929

[42] Foldi J Kahn A Silber A Qing T Reisenbichler E Fischbach N. Clinical outcomes and immune markers by race in a phase I/II clinical trial of durvalumab concomitant with neoadjuvant chemotherapy in early-stage TNBC. Clin Cancer Res (2022) 28(17):3720–8. doi: 10.1158/1078-0432.CCR-22-0862 PMC9444984 35903931

[43] Rathmell JC. Obesity, immunity, and cancer. N Engl J Med (2021) 384(12):1160–2. doi: 10.1056/NEJMcibr2035081 PMC8053443 33761213

[44] An Y Wu Z Wang N Yang Z Li Y Xu B. Association between body mass index and survival outcomes for cancer patients treated with immune checkpoint inhibitors: a systematic review and meta-analysis. J Transl Med (2020) 18(1):235. doi: 10.1186/s12967-020-02404-x 32532255 PMC7291531

[45] Kichenadasse G Miners JO Mangoni AA Rowland A Hopkins AM Sorich MJ. Association between body mass index and overall survival with immune checkpoint inhibitor therapy for advanced non-small cell lung cancer. JAMA Oncol (2020) 6(4):512–8. doi: 10.1001/jamaoncol.2019.5241 PMC6990855 31876896

[46] Roh J Eom JS Lee MK Kim J Jang T Yoon SH. Prognostic factors of second-line immune checkpoint inhibitors in patients with advanced-stage non-small cell lung cancer: a multicenter, retrospective study. Am J Clin Oncol (2021) 44(7):356–60. doi: 10.1097/COC.0000000000000828 34014843

[47] McQuade JL Daniel CR Hess KR Mak C Wang DY Rai RR. Association of body-mass index and outcomes in patients with metastatic melanoma treated with targeted therapy, immunotherapy, or chemotherapy: a retrospective, multicohort analysis. Lancet Oncol (2018) 19(3):310–22. doi: 10.1016/S1470-2045(18)30078-0 PMC5840029 29449192

[48] Ichihara E Harada D Inoue K Sato K Hosokawa S Kishino D. The impact of body mass index on the efficacy of anti-PD-1/PD-L1 antibodies in patients with non-small cell lung cancer. Lung Canc (2020) 139:140–5. doi: 10.1016/j.lungcan.2019.11.011 31786476

[49] Biswas AK Acharyya S. Understanding cachexia in the context of metastatic progression. Nat Rev Canc (2020) 20(5):274–84. doi: 10.1038/s41568-020-0251-4 32235902

[50] Joshi I Peravali M Geng X Rao S Chen KY Veytsman I. Impact of baseline clinical biomarkers on treatment outcomes in patients with advanced NSCLC receiving first-line pembrolizumab-based therapy. Clin Lung Cancer (2022) 23(5):438–45. doi: 10.1016/j.cllc.2022.03.010 35649819

[51] Jo H Yoshida T Horinouchi H Yagishita S Matsumoto Y Shinno Y. Prognostic significance of cachexia in advanced non-small cell lung cancer patients treated with pembrolizumab. Cancer Immunol Immunother (2022) 71(2):387–98. doi: 10.1007/s00262-021-02997-2 PMC10991209 34180007

[52] You Y Jiang C Peng K He W Wang L Jin Y. The predictive value of body mass index on prognosis and adverse events of cancers treated with immunotherapy: a systematic review and meta-analysis. Cancer Immunol Immunother. (2021) 70(8):2323–35. doi: 10.1007/s00262-021-02858-y PMC10991956 33512554

[53] Cortellini A Bersanelli M Santini D Buti S Tiseo M Cannita K. Another side of the association between body mass index (BMI) and clinical outcomes of cancer patients receiving programmed cell death protein-1 (PD-1)/Programmed cell death-ligand 1 (PD-L1) checkpoint inhibitors: a multicentre analysis of immune-related adverse events. Eur J Canc (2020) 128:17–26. doi: 10.1016/j.ejca.2019.12.031 32109847

[54] Dos Santos DMC Rejeski K Winkelmann M Liu L Trinkner P Günther S. Increased visceral fat distribution and body composition impact cytokine release syndrome onset and severity after CD19 chimeric antigen receptor T-cell therapy in advanced b-cell malignancies. Haematologica (2022) 107(9):2096–107. doi: 10.3324/haematol.2021.280189 PMC9425325 35172565

[55] Minami S Ihara S Tanaka T Komuta K. Sarcopenia and visceral adiposity did not affect efficacy of immune-checkpoint inhibitor monotherapy for pretreated patients with advanced non-small cell lung cancer. World J Oncol (2020) 11(1):9–22. doi: 10.14740/wjon1225 32095185 PMC7011908

[56] Bolte FJ McTavish S Wakefield N Shantzer L Hubbard C Krishnaraj A. Association of sarcopenia with survival in advanced NSCLC patients receiving concurrent immunotherapy and chemotherapy. Front Oncol (2022) 12:986236. doi: 10.3389/fonc.2022.986236 36212442 PMC9539742

[57] Bessede A Marabelle A Guégan JP Danlos FX Cousin S Peyraud F. Impact of acetaminophen on the efficacy of immunotherapy in cancer patients. Ann Oncol (2022) 33(9):909–15. doi: 10.1016/j.annonc.2022.05.010 35654248

[58] Lurienne L Cervesi J Duhalde L de Gunzburg J Andremont A Zalcman G. NSCLC immunotherapy efficacy and antibiotic use: a systematic review and meta-analysis. J Thorac Oncol (2020) 15(7):1147–59. doi: 10.1016/j.jtho.2020.03.002 32173463

[59] Spencer CN McQuade JL Gopalakrishnan V McCulloch JA Vetizou M Cogdill AP. Dietary fiber and probiotics influence the gut microbiome and melanoma immunotherapy response. Science (2021) 374(6575):1632–40. doi: 10.1126/science.aaz7015 PMC8970537 34941392

[60] Tomita Y Ikeda T Sakata S Saruwatari K Sato R Iyama S. Association of probiotic clostridium butyricum therapy with survival and response to immune checkpoint blockade in patients with lung cancer. Cancer Immunol Res (2020) 8(10):1236–42. doi: 10.1158/2326-6066.CIR-20-0051 32665261

[61] Matson V Chervin CS Gajewski TF. Cancer and the microbiome–influence of the commensal microbiota on cancer, immune responses, and immunotherapy. Gastroenterology (2021) 160(2):600–13. doi: 10.1053/j.gastro.2020.11.041 PMC8409239 33253684

[62] Vétizou M Pitt JM Daillère R Lepage P Waldschmitt N Flament C. Anticancer immunotherapy by CTLA-4 blockade relies on the gut microbiota. Science (2015) 350(6264):1079–84. doi: 10.1126/science.aad1329 PMC4721659 26541610

[63] Sivan A Corrales L Hubert N Williams JB Aquino-Michaels K Earley ZM. Commensal bifidobacterium promotes antitumor immunity and facilitates anti-PD-L1 efficacy. Science (2015) 350(6264):1084–9. doi: 10.1126/science.aac4255 PMC4873287 26541606

[64] Uribe-Herranz M Bittinger K Rafail S Guedan S Pierini S Tanes C. Gut microbiota modulates adoptive cell therapy via CD8α dendritic cells and IL-12. JCI Insight (2018) 3(4):e94952. doi: 10.1172/jci.insight.94952 29467322 PMC5916241

[65] Hakozaki T Richard C Elkrief A Hosomi Y Benlaïfaoui M Mimpen I. The gut microbiome associates with immune checkpoint inhibition outcomes in patients with advanced non–small cell lung cancer. Cancer Immunol Res (2020) 8(10):1243–50. doi: 10.1158/2326-6066.CIR-20-0196 32847937

[66] Andrews MC Duong CPM Gopalakrishnan V Iebba V Chen WS Derosa L. Gut microbiota signatures are associated with toxicity to combined CTLA-4 and PD-1 blockade. Nat Med (2021) 27(8):1432–41. doi: 10.1038/s41591-021-01406-6 PMC11107795 34239137

[67] Routy B Le Chatelier E Derosa L Duong CPM Alou MT Daillère R. Gut microbiome influences efficacy of PD-1-based immunotherapy against epithelial tumors. Science (2018) 359(6371):91–7. doi: 10.1126/science.aan3706 29097494

[68] Baruch EN Youngster I Ben-Betzalel G Ortenberg R Lahat A Katz L. Fecal microbiota transplant promotes response in immunotherapy-refractory melanoma patients. Science (2021) 371(6529):602–9. doi: 10.1126/science.abb5920 33303685

[69] Fuhler GM. The immune system and microbiome in pregnancy. Best Pract Res Clin Gastroenterol (2020) 44–45:101671. doi: 10.1016/j.bpg.2020.101671 32359685

[70] Mor G Cardenas I Abrahams V Guller S. Inflammation and pregnancy: the role of the immune system at the implantation site. Ann N Y Acad Sci (2011) 1221(1):80–7. doi: 10.1111/j.1749-6632.2010.05938.x PMC3078586 21401634

[71] Yang F Zheng Q Jin L. Dynamic function and composition changes of immune cells during normal and pathological pregnancy at the maternal-fetal interface. Front Immunol (2019) 10:2317. doi: 10.3389/fimmu.2019.02317 31681264 PMC6813251

[72] Zhao SJ Muyayalo KP Luo J Huang D Mor G Liao AH. Next generation of immune checkpoint molecules in maternal-fetal immunity. Immunol Rev (2022) 308(1):40–54. doi: 10.1111/imr.13073 35234305

[73] Veras E Kurman RJ Wang TL Shih IM. PD-L1 expression in human placentas and gestational trophoblastic diseases. Int J Gynecol Pathol (2017) 36(2):146–53. doi: 10.1097/PGP.0000000000000305 PMC5518625 27362903

[74] Wang S Sun F Li M Qian J Chen C Wang M. The appropriate frequency and function of decidual Tim-3+CTLA-4+CD8+ T cells are important in maintaining normal pregnancy. Cell Death Dis (2019) 10(6):407. doi: 10.1038/s41419-019-1642-x 31138782 PMC6538701

[75] Zhao Y Zheng Q Jin L. The role of B7 family molecules in maternal-fetal immunity. Front Immunol (2020) 11:458. doi: 10.3389/fimmu.2020.00458 32265918 PMC7105612

[76] Salehi I Porto L Elser C Singh J Saibil S Maxwell C. Immune checkpoint inhibitor exposure in pregnancy: a scoping review. J Immunother (2022) 45(5):231. doi: 10.1097/CJI.0000000000000418 35353074 PMC9087868

[77] Xu W Moor RJ Walpole ET Atkinson VG. Pregnancy with successful foetal and maternal outcome in a melanoma patient treated with nivolumab in the first trimester: case report and review of the literature. Melanoma Res (2019) 29(3):333–7. doi: 10.1097/CMR.0000000000000586 30730328

[78] Bucheit AD Hardy JT Szender JB Glitza Oliva IC. Conception and viable twin pregnancy in a patient with metastatic melanoma while treated with CTLA-4 and PD-1 checkpoint inhibition. Melanoma Res (2020) 30(4):423–5. doi: 10.1097/CMR.0000000000000657 32073510

[79] Haiduk J Ziemer M. Pregnancy in a patient with metastatic uveal melanoma treated with nivolumab. J Dtsch Dermatol Ges. (2021) 19(5):762–5. doi: 10.1111/ddg.14463 33768686

[80] Burotto M Gormaz JG Samtani S Valls N Silva R Rojas C. Viable pregnancy in a patient with metastatic melanoma treated with double checkpoint immunotherapy. Semin Oncol (2018) 45(3):164–9. doi: 10.1053/j.seminoncol.2018.03.003 30262400

[81] Polnaszek B Mullen M Bligard K Raghuraman N Massad LS. Term pregnancy after complete response of placental site trophoblastic tumor to immunotherapy. Obstet Gynecol. (2021) 138(1):115–8. doi: 10.1097/AOG.0000000000004434 34259474

[82] Menzer C Beedgen B Rom J Duffert CM Volckmar AL Sedlaczek O. Immunotherapy with ipilimumab plus nivolumab in a stage IV melanoma patient during pregnancy. Eur J Canc (2018) 104:239–42. doi: 10.1016/j.ejca.2018.09.008 30454709

[83] Mehta A Kim KB Minor DR. Case report of a pregnancy during ipilimumab therapy. J Glob Oncol (2018) 4:1–3. doi: 10.1200/JGO.17.00019 PMC6180753 30241195

[84] Stang A Schwärzler P Schmidtke S Tolosa E Kobbe R. Successful immunochemotherapy for burkitt lymphoma during pregnancy as a bridge to postpartum high-dose methotrexate therapy: a case report and review of the literature. Clin Lymphoma Myeloma Leukemia (2020) 20(6):e284–90. doi: 10.1016/j.clml.2019.12.012 32165154

[85] Barbur E Doganca T Obek C. Safe use of intravesical bacillus calmette-guérin immunotherapy for bladder cancer during breastfeeding: a case report. Immunotherapy (2022) 14(10):759–64. doi: 10.2217/imt-2021-0203 35754395

[86] Plummer M de Martel C Vignat J Ferlay J Bray F Franceschi S. Global burden of cancers attributable to infections in 2012: a synthetic analysis. Lancet Glob Health (2016) 4(9):e609–616. doi: 10.1016/S2214-109X(16)30143-7 27470177

[87] Mesri EA Feitelson MA Munger K. Human viral oncogenesis: a cancer hallmarks analysis. Cell Host Microbe (2014) 15(3):266–82. doi: 10.1016/j.chom.2014.02.011 PMC3992243 24629334

[88] Ilhan ZE Łaniewski P Thomas N Roe DJ Chase DM Herbst-Kralovetz MM. Deciphering the complex interplay between microbiota, HPV, inflammation and cancer through cervicovaginal metabolic profiling. EBioMedicine (2019) 44:675–90. doi: 10.1016/j.ebiom.2019.04.028 PMC6604110 31027917

[89] Moir S Chun TW Fauci AS. Pathogenic mechanisms of HIV disease. Annu Rev Pathol (2011) 6:223–48. doi: 10.1146/annurev-pathol-011110-130254 21034222

[90] Wang R Pan W Jin L Huang W Li Y Wu D. Human papillomavirus vaccine against cervical cancer: opportunity and challenge. Cancer Lett (2020) 471:88–102. doi: 10.1016/j.canlet.2019.11.039 31812696

[91] Draper LM Kwong MLM Gros A Stevanović S Tran E Kerkar S. Targeting of HPV-16+ epithelial cancer cells by TCR gene engineered T cells directed against E6. Clin Cancer Res (2015) 21(19):4431–9. doi: 10.1158/1078-0432.CCR-14-3341 PMC4603283 26429982

[92] Magazine N Zhang T Wu Y McGee MC Veggiani G Huang W. Mutations and evolution of the SARS-CoV-2 spike protein. Viruses (2022) 14(3):640. doi: 10.3390/v14030640 35337047 PMC8949778

[93] Jamal M Bangash HI Habiba M Lei Y Xie T Sun J. Immune dysregulation and system pathology in COVID-19. Virulence (2021) 12(1):918–36. doi: 10.1080/21505594.2021.1898790 PMC7993139 33757410

[94] Williamson EJ Walker AJ Bhaskaran K Bacon S Bates C Morton CE. Factors associated with COVID-19-related death using OpenSAFELY. Nature (2020) 584(7821):430–6. doi: 10.1038/s41586-020-2521-4 PMC7611074 32640463

[95] Wu H Liao S Wang Y Guo M Lin X Wu J. Molecular evidence suggesting the persistence of residual SARS-CoV-2 and immune responses in the placentas of pregnant patients recovered from COVID-19. Cell Prolif. (2021) 54(9):e13091. doi: 10.1111/cpr.13091 34291856 PMC8420381

[96] Lv D Hu B Lin X Wang R Wu D Long R. Immunopathogenesis of patients with COVID-19: from the perspective of immune system “evolution” and “revolution” Expert Rev Mol Med (2022) 24:e19. doi: 10.1017/erm.2022.12 35535759 PMC9884756

[97] Fathi M Vakili K Jazi K Sadeghi MA Hajiesmaeili M Mohamadkhani A. Challenges of cancer immunotherapy and chemotherapy during the COVID-19 pandemic. Tumori (2022) 108(5):407–19. doi: 10.1177/03008916211063939 34918602

[98] Fendler A de Vries EGE GeurtsvanKessel CH Haanen JB Wörmann B Turajlic S. COVID-19 vaccines in patients with cancer: immunogenicity, efficacy and safety. Nat Rev Clin Oncol (2022) 19(6):385–401. doi: 10.1038/s41571-022-00610-8 35277694 PMC8916486

[99] Houot R Levy R Cartron G Armand P. Could anti-CD20 therapy jeopardise the efficacy of a SARS-CoV-2 vaccine? Eur J Cancer (2020) 136:4–6. doi: 10.1016/j.ejca.2020.06.017 PMC7315961 32619884

[100] Zeng C Evans JP Chakravarthy K Qu P Reisinger S Song NJ. COVID-19 mRNA booster vaccines elicit strong protection against SARS-CoV-2 omicron variant in patients with cancer. Cancer Cell (2022) 40(2):117–9. doi: 10.1016/j.ccell.2021.12.014 PMC8716174 34986328

[101] Dai M Liu D Liu M Zhou F Li G Chen Z. Patients with cancer appear more vulnerable to SARS-CoV-2: a multicenter study during the COVID-19 outbreak. Cancer Discovery (2020) 10(6):783–91. doi: 10.1158/2159-8290.CD-20-0422 PMC7309152 32345594

[102] von Lilienfeld-Toal M Vehreschild JJ Cornely O Pagano L Compagno F EHA Infectious Disease Scientific Working Group. Frequently asked questions regarding SARS-CoV-2 in cancer patients-recommendations for clinicians caring for patients with malignant diseases. Leukemia (2020) 34(6):1487–94. doi: 10.1038/s41375-020-0832-y PMC7194246 32358568

[103] Bersanelli M Buti S Banna GL De Giorgi U Cortellini A Rebuzzi SE. Impact of influenza syndrome and flu vaccine on survival of cancer patients during immunotherapy in the INVIDIa study. Immunotherapy (2020) 12(2):151–9. doi: 10.2217/imt-2019-0180 32089035

[104] Yatim N Boussier J Tetu P Smith N Bruel T Charbit B. Immune checkpoint inhibitors increase T cell immunity during SARS-CoV-2 infection. Sci Adv (2021) 7(34):eabg4081. doi: 10.1126/sciadv.abg4081 34407944 PMC8373136

[105] Yarza R Bover M Paredes D López-López F Jara-Casas D Castelo-Loureiro A. SARS-CoV-2 infection in cancer patients undergoing active treatment: analysis of clinical features and predictive factors for severe respiratory failure and death. Eur J Canc (2020) 135:242–50. doi: 10.1016/j.ejca.2020.06.001 PMC7275164 32586724

[106] Qin C Zhou L Hu Z Zhang S Yang S Tao Y. Dysregulation of immune response in patients with coronavirus 2019 (COVID-19) in wuhan, China. Clin Infect Dis (2020) 71(15):762–8. doi: 10.1093/cid/ciaa248 PMC7108125 32161940

[107] Lim SY Lee JH Gide TN Menzies AM Guminski A Carlino MS. Circulating cytokines predict immune-related toxicity in melanoma patients receiving anti-PD-1-Based immunotherapy. Clin Cancer Res (2019) 25(5):1557–63. doi: 10.1158/1078-0432.CCR-18-2795 30409824

[108] Cheng YK Chen P Chen DW Lin ZS Ye SB Lan P. Comparative safety, efficacy and survival outcome of anti-PD-1 immunotherapy in colorectal cancer patients with vs without hepatitis b virus infection: a multicenter cohort study. Clin Transl Gastroenterol (2022) 13(5):e00475. doi: 10.14309/ctg.0000000000000475 35297794 PMC9132513

[109] Shah NJ Al-Shbool G Blackburn M Cook M Belouali A Liu SV. Safety and efficacy of immune checkpoint inhibitors (ICIs) in cancer patients with HIV, hepatitis b, or hepatitis c viral infection. J Immunother Canc (2019) 7(1):353. doi: 10.1186/s40425-019-0771-1 PMC6918622 31847881

[110] Puronen CE Ford ES Uldrick TS. Immunotherapy in people with HIV and cancer. Front Immunol (2019) 10:2060. doi: 10.3389/fimmu.2019.02060 31555284 PMC6722204

[111] Uldrick TS Gonçalves PH Abdul-Hay M Claeys AJ Emu B Ernstoff MS. Assessment of the safety of pembrolizumab in patients with HIV and advanced cancer-a phase 1 study. JAMA Oncol (2019) 5(9):1332–9. doi: 10.1001/jamaoncol.2019.2244 PMC6547135 31154457

[112] Le DT Durham JN Smith KN Wang H Bartlett BR Aulakh LK. Mismatch repair deficiency predicts response of solid tumors to PD-1 blockade. Science (2017) 357(6349):409–13. doi: 10.1126/science.aan6733 PMC5576142 28596308

[113] Chung HC Ros W Delord JP Perets R Italiano A Shapira-Frommer R. Efficacy and safety of pembrolizumab in previously treated advanced cervical cancer: results from the phase II KEYNOTE-158 study. J Clin Oncol (2019) 37(17):1470–8. doi: 10.1200/JCO.18.01265 30943124

[114] De Mattos-Arruda L Vazquez M Finotello F Lepore R Porta E Hundal J. Neoantigen prediction and computational perspectives towards clinical benefit: recommendations from the ESMO precision medicine working group. Ann Oncol (2020) 31(8):978–90. doi: 10.1016/j.annonc.2020.05.008 PMC7885309 32610166

[115] Ri M Iida S Maruyama D Sakabe A Kamei R Nakashima T. HLA genotyping in Japanese patients with multiple myeloma receiving bortezomib: an exploratory biomarker study of JCOG1105 (JCOG1105A1). Cancer Sci (2021) 112(12):5011–9. doi: 10.1111/cas.15158 PMC8645746 34626515

[116] Chowell D Morris LGT Grigg CM Weber JK Samstein RM Makarov V. Patient HLA class I genotype influences cancer response to checkpoint blockade immunotherapy. Science (2018) 359(6375):582–7. doi: 10.1126/science.aao4572 PMC6057471 29217585

[117] Lee CH DiNatale RG Chowell D Krishna C Makarov V Valero C. High response rate and durability driven by HLA genetic diversity in patients with kidney cancer treated with lenvatinib and pembrolizumab. Mol Cancer Res (2021) 19(9):1510–21. doi: 10.1158/1541-7786.MCR-21-0053 PMC8419018 34039647

[118] Shindo Y Hazama S Tsunedomi R Suzuki N Nagano H. Novel biomarkers for personalized cancer immunotherapy. Cancers (Basel). (2019) 11(9):E1223. doi: 10.3390/cancers11091223 PMC6770350 31443339

[119] Joyce JA Fearon DT. T Cell exclusion, immune privilege, and the tumor microenvironment. Science (2015) 348(6230):74–80. doi: 10.1126/science.aaa6204 25838376

[120] James FR Jiminez-Linan M Alsop J Mack M Song H Brenton JD. Association between tumour infiltrating lymphocytes, histotype and clinical outcome in epithelial ovarian cancer. BMC Canc (2017) 17(1):657. doi: 10.1186/s12885-017-3585-x PMC5607562 28931370

[121] Hsu CL Ou DL Bai LY Chen CW Lin L Huang SF. Exploring markers of exhausted CD8 T cells to predict response to immune checkpoint inhibitor therapy for hepatocellular carcinoma. Liver Canc (2021) 10(4):346–59. doi: 10.1159/000515305 PMC8339511 34414122

[122] Holm JS Funt SA Borch A Munk KK Bjerregaard AM Reading JL. Neoantigen-specific CD8 T cell responses in the peripheral blood following PD-L1 blockade might predict therapy outcome in metastatic urothelial carcinoma. Nat Commun (2022) 13(1):1935. doi: 10.1038/s41467-022-29342-0 35410325 PMC9001725

[123] Kim JH Ryu MH Park YS Ma J Lee SY Kim D. Predictive biomarkers for the efficacy of nivolumab as ≥ 3rd-line therapy in patients with advanced gastric cancer: a subset analysis of ATTRACTION-2 phase III trial. BMC Canc (2022) 22(1):378. doi: 10.1186/s12885-022-09488-2 PMC8994342 35397540

[124] Liu Q Wang Y Gao H Sun F Wang X Zhang H. An individualized prognostic signature for clinically predicting the survival of patients with bladder cancer. Front Genet (2022) 13:837301. doi: 10.3389/fgene.2022.837301 35422849 PMC9002098

[125] Huynh E Hosny A Guthier C Bitterman DS Petit SF Haas-Kogan DA. Artificial intelligence in radiation oncology. Nat Rev Clin Oncol (2020) 17(12):771–81. doi: 10.1038/s41571-020-0417-8 32843739

[126] Contreras I Vehi J. Artificial intelligence for diabetes management and decision support: literature review. J Med Internet Res (2018) 20(5):e10775. doi: 10.2196/10775 29848472 PMC6000484

[127] Ting DSW Pasquale LR Peng L Campbell JP Lee AY Raman R. Artificial intelligence and deep learning in ophthalmology. Br J Ophthalmol (2019) 103(2):167–75. doi: 10.1136/bjophthalmol-2018-313173 PMC6362807 30361278

[128] Akselrod-Ballin A Chorev M Shoshan Y Spiro A Hazan A Melamed R. Predicting breast cancer by applying deep learning to linked health records and mammograms. Radiology (2019) 292(2):331–42. doi: 10.1148/radiol.2019182622 31210611

[129] Ardila D Kiraly AP Bharadwaj S Choi B Reicher JJ Peng L. End-to-end lung cancer screening with three-dimensional deep learning on low-dose chest computed tomography. Nat Med (2019) 25(6):954–61. doi: 10.1038/s41591-019-0447-x 31110349

[130] Esteva A Kuprel B Novoa RA Ko J Swetter SM Blau HM. Dermatologist-level classification of skin cancer with deep neural networks. Nature (2017) 542(7639):115–8. doi: 10.1038/nature21056 PMC8382232 28117445

[131] Chen H Yang BW Qian L Meng YS Bai XH Hong XW. Deep learning prediction of ovarian malignancy at US compared with O-RADS and expert assessment. Radiology (2022) 304(1):106–13. doi: 10.1148/radiol.211367 35412367

[132] Wang S Liu Z Rong Y Zhou B Bai Y Wei W. Deep learning provides a new computed tomography-based prognostic biomarker for recurrence prediction in high-grade serous ovarian cancer. Radiother Oncol (2019) 132:171–7. doi: 10.1016/j.radonc.2018.10.019 30392780

[133] Li B Qi J Fu L Han J Choo J deMello AJ. Integrated hand-powered centrifugation and paper-based diagnosis with blood-in/answer-out capabilities. Biosens Bioelectron. (2020) 165:112282. doi: 10.1016/j.bios.2020.112282 32729467

[134] Ahmed A Aziz S Abd-Alrazaq A Farooq F Sheikh J. Overview of artificial intelligence-driven wearable devices for diabetes: scoping review. J Med Internet Res (2022) 24(8):e36010. doi: 10.2196/36010 35943772 PMC9399882

[135] Wu J Liu C Liu X Sun W Li L Gao N. Artificial intelligence-assisted system for precision diagnosis of PD-L1 expression in non-small cell lung cancer. Mod Pathol (2022) 35(3):403–11. doi: 10.1038/s41379-021-00904-9 34518630

[136] Shamai G Livne A Polónia A Sabo E Cretu A Bar-Sela G. Deep learning-based image analysis predicts PD-L1 status from H&E-stained histopathology images in breast cancer. Nat Commun (2022) 13(1):6753. doi: 10.1038/s41467-022-34275-9 36347854 PMC9643479

[137] Kapil A Meier A Zuraw A Steele KE Rebelatto MC Schmidt G. Deep semi supervised generative learning for automated tumor proportion scoring on NSCLC tissue needle biopsies. Sci Rep (2018) 8(1):17343. doi: 10.1038/s41598-018-35501-5 30478349 PMC6255873

[138] Trebeschi S Drago SG Birkbak NJ Kurilova I Cǎlin AM Delli Pizzi A. Predicting response to cancer immunotherapy using noninvasive radiomic biomarkers. Ann Oncol (2019) 30(6):998–1004. doi: 10.1093/annonc/mdz108 30895304 PMC6594459

[139] Abbasi J. “Electronic nose” predicts immunotherapy response. JAMA (2019) 322(18):1756. doi: 10.1001/jama.2019.18225 31714968

[140] Kleine-Kohlbrecher D Petersen NV Pavlidis MA Trolle T Friis S Kringelum J. Abstract LB199: a personalized neoantigen vaccine is well tolerated and induces specific T-cell immune response in patients with resected melanoma. Cancer Res (2023) 83(8_Supplement):LB199. doi: 10.1158/1538-7445.AM2023-LB199

[141] Chowell D Yoo SK Valero C Pastore A Krishna C Lee M. Improved prediction of immune checkpoint blockade efficacy across multiple cancer types. Nat Biotechnol (2022) 40(4):499–506. doi: 10.1038/s41587-021-01070-8 34725502 PMC9363980

[142] Barrera C Corredor G Viswanathan VS Ding R Toro P Fu P. Deep computational image analysis of immune cell niches reveals treatment-specific outcome associations in lung cancer. NPJ Precis Oncol (2023) 7(1):52. doi: 10.1038/s41698-023-00403-x 37264091 PMC10235089

[143] Houy N Le Grand F. Optimizing immune cell therapies with artificial intelligence. J Theor Biol (2019) 461:34–40. doi: 10.1016/j.jtbi.2018.09.007 30352236

[144] Charoentong P Finotello F Angelova M Mayer C Efremova M Rieder D. Pan-cancer immunogenomic analyses reveal genotype-immunophenotype relationships and predictors of response to checkpoint blockade. Cell Rep (2017) 18(1):248–62. doi: 10.1016/j.celrep.2016.12.019 28052254

[145] Hindocha S Charlton TG Linton-Reid K Hunter B Chan C Ahmed M. A comparison of machine learning methods for predicting recurrence and death after curative-intent radiotherapy for non-small cell lung cancer: development and validation of multivariable clinical prediction models. EBioMedicine (2022) 77:103911. doi: 10.1016/j.ebiom.2022.103911 35248997 PMC8897583

